# A prognostic model for long-term outcome in moderate-severe traumatic brain injury using multimodal neuromonitoring: a retrospective cohort analysis

**DOI:** 10.3389/fneur.2025.1708954

**Published:** 2026-08-26

**Authors:** Yang Li, Kanglin Liu, XiaoPing He, Yixin Lin, Yuan Ma

**Affiliations:** 1 Department of Neurosurgery, The General Hospital of Western Theater Command, Chengdu, Sichuan, China; 2 Pu'er People's Hospital, Pu'er, China; 3 Beijing Chaoyang Hospital Affiliated to Capital Medical University, Beijing, China

**Keywords:** traumatic brain injury, multimodal monitoring, prognostic model, brain tissue oxygen, lactate/pyruvate ratio, Glasgow Outcome Scale-Extended

## Abstract

**Background:**

Moderate-to-severe traumatic brain injury (msTBI) remains a leading cause of long-term disability and mortality. While multimodal neuromonitoring (MMM) provides real-time insights into cerebral pathophysiology, its integration into prognostic models for long-term outcomes requires further validation.

**Objective:**

To develop and evaluate a prognostic model for 6-month neurological outcomes in msTBI patients using acute-phase multimodal monitoring parameters, clinical variables, and comorbidities.

**Methods:**

We conducted a retrospective cohort study of 143 adult patients with msTBI [Glasgow Coma Scale (GCS) 4–12] who underwent multimodal intracranial monitoring (ICP, CPP, PbtO₂, and cerebral microdialysis) for ≥48 h at *Hospital* between June 2022 and May 2025. Demographic, clinical, and monitoring data from the first 5 days were collected. The primary outcome was neurological function at 6 months, assessed by the Glasgow Outcome Scale-Extended (GOSE), dichotomized as favorable (GOSE 5–8) or unfavorable (GOSE 1–4). Multivariable logistic regression was used to identify independent predictors, and a prognostic model was constructed and internally validated using ROC analysis and the Hosmer-Lemeshow test.

**Results:**

Of 143 patients, 86 (60.1%) had favorable outcomes and 57 (39.9%) had unfavorable outcomes (including 29 deaths). Independent predictors of unfavorable outcome were: older age (adjusted OR 1.055 per year, 95% CI: 1.015–1.098), lower admission GCS (OR 0.713, 95% CI: 0.537–0.947), elevated lactate/pyruvate ratio (LPR) (OR 1.129 per unit, 95% CI: 1.044–1.220), reduced brain tissue oxygen tension (PbtO₂) (OR 0.842 per mmHg, 95% CI: 0.730–0.972), and pre-existing coronary heart disease (OR 3.866, 95% CI: 0.998–14.976). The final model demonstrated excellent discriminative performance with an AUC of 0.882 (95% CI: 0.820–0.944) and good calibration (Hosmer-Lemeshow *p* = 0.523). A dose–response relationship was observed between the number of abnormal monitoring parameters and risk of poor outcome.

**Conclusion:**

A prognostic model incorporating multimodal neuromonitoring parameters (LPR and PbtO₂), age, admission GCS, and coronary heart disease accurately predicts 6-month outcomes in msTBI patients. These findings support the clinical utility of integrated neuromonitoring for early risk stratification and personalized neurocritical care.

## 1 Introduction

Moderate–severe traumatic brain injury (msTBI) is one of the leading causes of mortality and long-term disability worldwide, imposing a substantial burden on patients, families, and healthcare systems (1, 2). The pathophysiology of msTBI involves not only the primary injury but, more critically, a cascade of secondary brain injuries. These are often driven by complex mechanisms such as cerebral edema, elevated intracranial pressure (ICP), reduced cerebral perfusion pressure (CPP), and cerebral hypoxia, which are central to neurological deterioration and poor outcomes (3–5).

Conventional neurocritical care has largely relied on ICP and CPP monitoring to guide treatment. However, growing evidence suggests that management based solely on ICP/CPP parameters has limitations and may not fully reflect the metabolic and oxygenation status of the brain tissue (6–8). In response, multimodal monitoring (MMM) has emerged as an advanced approach in modern TBI care. By integrating technologies such as brain tissue oxygenation (PbtO₂), cerebral microdialysis (MD) [which provides metabolic markers like the lactate/pyruvate ratio (LPR)], and continuous electroencephalography (cEEG), MMM aims to detect secondary pathophysiological changes earlier and more accurately, thereby supporting individualized and precise treatment strategies (8–10).

Despite the theoretical advantages of multimodal monitoring, its widespread clinical adoption faces several challenges. These include determining the optimal combination of monitoring parameters, defining clinically relevant thresholds, and translating complex data into actionable interventions that improve patient outcomes—all of which require further high-quality clinical evidence (10–12).

This study retrospectively analyzed acute-phase multimodal monitoring data from patients with msTBI treated at hospital. We examined the association between four key parameters—ICP, CPP, PbtO₂, and LPR—and long-term neurological outcomes. Furthermore, we developed a prognostic model based on multimodal data. Our findings support the hypothesis that the integration of multimodal monitoring parameters significantly enhanced the accuracy of predicting unfavorable outcomes in msTBI patients, thereby providing a robust evidence-based tool to support early clinical decision-making and personalized treatment.

## 2 Methods

### 2.1 Study design and participants

This study used a retrospective observational cohort design. We consecutively included all eligible patients with moderate-to-severe traumatic brain injury (msTBI) admitted to *Hospital*, affiliated with Capital Medical University, between June 1, 2022, and May 1, 2024. Inclusion criteria were as follows: (1) Age ≥ 18 years; (2) Diagnosis of msTBI, defined as a Glasgow Coma Scale (GCS) score between 4 and 12 within 24 h after injury; (3) Receipt of standardized multimodal intracranial monitoring after admission. This included ICP monitoring plus at least one additional monitoring modality (such as PbtO₂ or cerebral microdialysis), with continuous monitoring for ≥ 48 h. Exclusion criteria included: (1) Penetrating or open brain injuries (e.g., gunshot wounds, stab wounds, open skull fractures with exposed brain tissue); (2) Pre-existing severe neurological dysfunction, defined as a modified Rankin Scale (mRS) score > 2; (3) Life-threatening polytrauma meeting any of the following: ① Injury Severity Score (ISS) > 25; ② Hemorrhagic shock (systolic blood pressure < 90 mmHg for > 30 min or requiring ≥ 4 units of red blood cell transfusion); ③ Severe thoracic or abdominal organ injury (Abbreviated Injury Scale [AIS] score ≥ 3) requiring emergency surgery; (4) Signs of brain death on admission or early stage, or receipt of palliative care/withdrawal of active treatment within 24 h after monitoring initiation; (5) Severe metabolic disorders or systemic diseases that could interfere with cerebral metabolism monitoring, including: ① Severe liver failure (Child-Pugh class C); ② Acute kidney injury requiring continuous renal replacement therapy (CRRT); ③ Uncontrolled thyroid storm or adrenal insufficiency; (6) Serious complications during monitor placement (e.g., intracranial hematoma requiring surgical evacuation) that could affect data reliability or outcomes; (7) Missing > 30% of multimodal monitoring data (calculated hourly over 5 consecutive days); (8) Loss to follow-up or incomplete key clinical outcome data; (9) Pregnancy (due to potential effects of physiological changes on cerebral perfusion and oxygenation parameters); (10) History of central nervous system diseases that could significantly affect prognosis or interpretation of monitoring, including: ① Post-stroke sequelae (mRS ≥ 2); ② History of severe traumatic brain injury with persistent neurological deficits; ③ Progressive neurodegenerative diseases (e.g., Alzheimer’s disease, Parkinson’s disease).

### 2.2 Data collection

#### 2.2.1 Baseline data

The following information was collected through the electronic medical record system: ① Demographic data: Age and sex. ② Injury characteristics: Mechanism of injury (e.g., traffic accident, fall, high-altitude fall). ③ Severity of injury: Glasgow Coma Scale (GCS) score on admission. ④ Medical history: Pre-existing comorbidities, including hypertension, diabetes, hyperlipidemia, and coronary artery disease.

#### 2.2.2 Multimodal monitoring data

All monitoring was performed according to institutional standard operating procedures. Data from the first 5 days after the start of monitoring were extracted from the bedside monitoring system: ① Intracranial pressure (ICP) and cerebral perfusion pressure (CPP): Continuously monitored using an intraparenchymal probe (e.g., Codman or Camino). The daily mean values were recorded. ② Brain tissue oxygen tension (PbtO₂): Monitored using a Licox or Neurovent-PTO probe. The daily mean values were recorded. ③ Cerebral microdialysis (MD): A CMA microdialysis system (MT100 + Brain Neurochemical Analysis System, ANATECH (Beijing) CO., LTD) was used. Samples were collected every 60 min. The lactate/pyruvate ratio (LPR) was analyzed, and the daily mean values were calculated. For analytical purposes, the above monitoring parameters were dichotomized according to widely accepted clinical thresholds: ICP > 20 mmHg; CPP < 60 mmHg; PbtO₂ < 20 mmHg; LPR > 25.

#### 2.2.3 Treatment measures

All targeted clinical interventions administered based on monitoring data during the study period were systematically recorded. These primarily included: (1) management of intracranial hypertension utilizing dehydrating agents and antihypertensive medications (e.g., mannitol, hypertonic saline); (2) sedation and analgesia therapies; and (3) secondary surgical interventions (e.g., decompressive craniectomy).

### 2.3 Outcome assessment

The primary outcome measure was neurological functional outcome at 6 months post-injury, assessed using the Glasgow Outcome Scale-Extended (GOSE) (13–15). Trained research personnel conducted the assessments during follow-up clinic visits or via telephone interviews. The GOSE scores range from 1 to 8, categorized as follows: Unfavorable Outcome: GOSE score 1–4 (including: 1. death; 2. vegetative state; 3–4. severe disability). Favorable Outcome: GOSE score 5–8 (including: 5–6. moderate disability; 7–8. good recovery). Based on the 6-month GOSE assessment, patients were dichotomized into two groups: the Favorable Outcome group (*n* = 86, 60.1%) and the Unfavorable Outcome group (*n* = 57, 39.9%).

### 2.4 Statistical analysis

All statistical analyses in this study were performed using R software (version 4.3.0) and GraphPad Prism (version 10.4). Missing data: For variables with <20% missingness, multiple imputation by chained equations (MICE) with 10 imputations was used. Complete-case analysis served as a sensitivity analysis and yielded consistent results. Descriptive Statistics: Continuous variables with normal distribution are presented as mean ± standard deviation (Mean ± SD), and comparisons between groups were conducted using the independent samples *t*-test. Continuous variables with non-normal distribution are expressed as median (interquartile range) [M (IQR)], and group comparisons were performed using the Mann–Whitney U test. Categorical data are presented as number (percentage) [*n* (%)], and comparisons between groups were made using the Chi-square test or Fisher’s exact test, as appropriate. Correlation Analysis: Pearson or Spearman correlation analysis was employed to explore the relationships between continuous monitoring parameters and the 6-month GOSE scores. Multivariate Analysis: Binary logistic regression (forward LR method) was used to identify independent risk factors for unfavorable outcome, with the 6-month prognosis (a binary variable: favorable = 0, unfavorable = 1) as the dependent variable. Variables with a *p*-value < 0.1 in univariate analysis, along with clinically relevant variables (e.g., comorbidities), were included in the multivariate model. Results are expressed as odds ratios (OR) with their corresponding 95% confidence intervals (CI). Prior to model construction, multicollinearity was evaluated using variance inflation factors (VIF). Variables with VIF > 5 were considered to have high collinearity. In this study, all VIF values were below 5, supporting the inclusion of all candidate variables in the multivariate model. Model Construction and Validation: A predictive model was constructed based on the results of the logistic regression analysis. The discriminative ability of the model was assessed by plotting the receiver operating characteristic (ROC) curve and calculating the area under the curve (AUC). The calibration of the model was evaluated using the Hosmer-Lemeshow goodness-of-fit test. Significance Level: A two-sided *p*-value < 0.05 was considered statistically significant.

## 3 Result

### 3.1 Patient baseline characteristics and outcome groups

**Figure 1:**
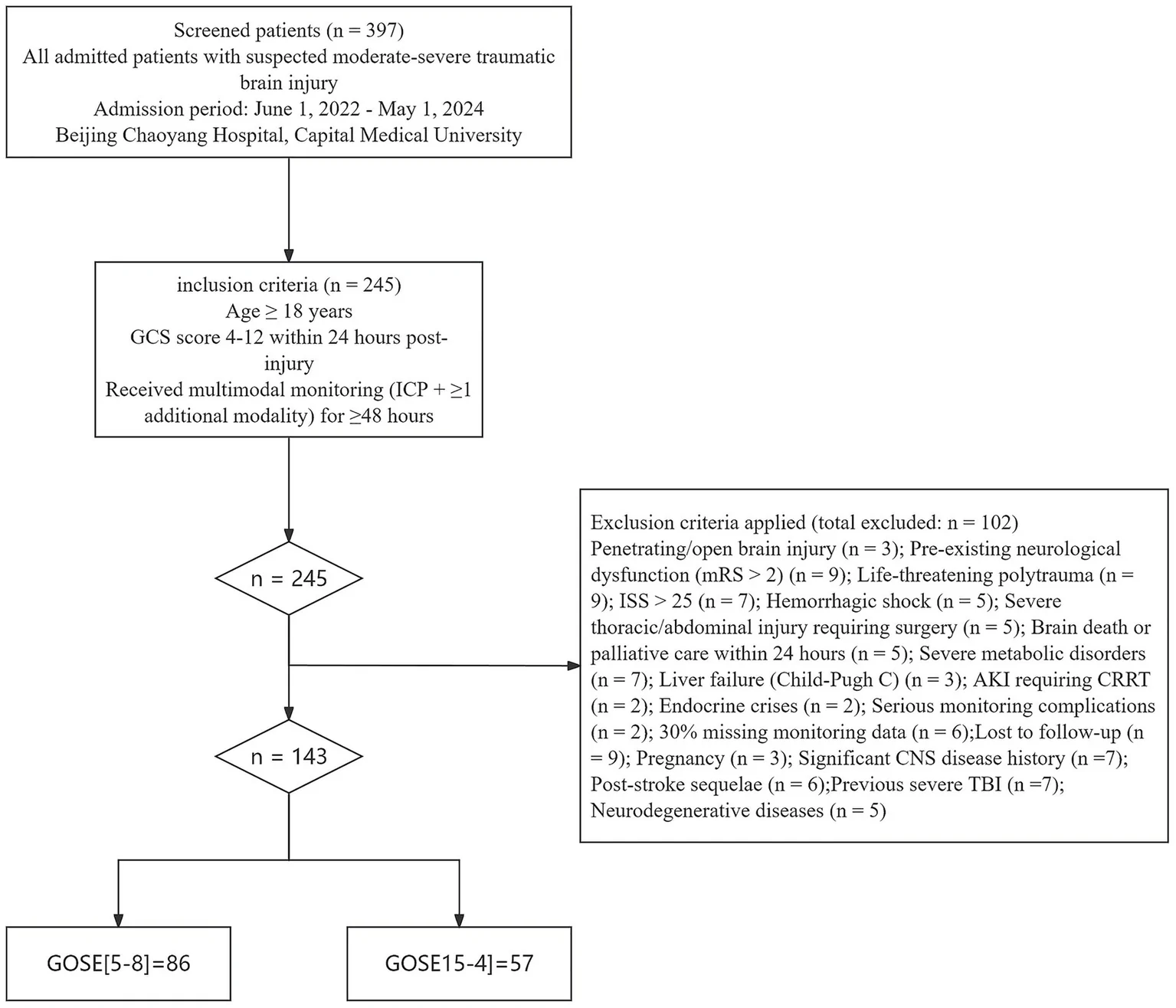
Flowchart of enrollment process.

A total of 143 patients with moderate-to-severe traumatic brain injury (msTBI) who met the inclusion criteria were ultimately enrolled in this study. Based on the Glasgow Outcome Scale-Extended (GOSE) scores at 6 months post-injury, patients were categorized into a favorable outcome group (GOSE 5–8, *n* = 86, 60.1%) and an unfavorable outcome group (GOSE 1–4, *n* = 57, 39.9%). Among these, 29 patients (20.3%) died. A comparison of the baseline characteristics between the two groups is presented in Table 1. Patients in the unfavorable outcome group were significantly older than those in the favorable outcome group (60.1 ± 16.3 years vs. 43.5 ± 15.2 years, *p* < 0.001) and had a higher prevalence of hypertension, diabetes, and coronary heart disease (all *p* < 0.05). No significant differences were observed between the two groups in terms of sex distribution or mechanisms of injury (see Figure 1).

**Table 1:** Comparison of baseline characteristics between patients with good and poor prognosis.

Characteristic	Total (*n* = 143)	Good prognosis (*n* = 86)	Poor prognosis (*n* = 57)	Statistic	*p*-value
Age (years), Mean ± SD	50.2 ± 17.8	43.5 ± 15.2	60.1 ± 16.3	*t* = −6.32	<0.001
Male, *n* (%)	84 (58.7)	53 (61.6)	31 (54.4)	*χ*^2^ = 0.76	0.382
Mechanism of Injury, *n* (%)				*χ*^2^ = 4.18	0.242
Traffic accident	72 (50.3)	48 (55.8)	24 (42.1)		
Fall	57 (39.9)	29 (33.7)	28 (49.1)		
Others	14 (9.8)	9 (10.5)	5 (8.8)		
Admission GCS, Mean ± SD	7.2 ± 2.3	8.1 ± 1.9	5.9 ± 2.1	*t* = 6.75	<0.001
Comorbidities, *n* (%)					
Hypertension	57 (39.9)	25 (29.1)	32 (56.1)	*χ*^2^ = 10.76	0.001
Diabetes	36 (25.2)	15 (17.4)	21 (36.8)	*χ*^2^ = 6.87	0.009
Hyperlipidemia	43 (30.1)	24 (27.9)	19 (33.3)	*χ*^2^ = 0.47	0.492
Coronary heart disease	22 (15.4)	7 (8.1)	15 (26.3)	*χ*^2^ = 8.93	0.003

### 3.2 Multimodal monitoring parameters and therapeutic interventions

Significant differences were observed in the acute-phase multimodal monitoring parameters between the two outcome groups (Table 2). The unfavorable outcome group demonstrated significantly higher ICP, lower CPP and PbtO₂, and elevated LPR compared to the favorable outcome group. Correspondingly, the proportion of patients receiving antihypertensive therapy, sedation/analgesia, and surgical interventions was significantly higher in the unfavorable outcome group (all *p* < 0.01).

**Table 2:** Comparison of multimodal monitoring parameters and clinical interventions.

Variable	Total (*n* = 143)	Good prognosis (*n* = 86)	Poor prognosis (*n* = 57)	Statistic	*p*-value
Monitoring Parameters, Mean ± SD					
ICP (mmHg)	20.1 ± 7.9	17.3 ± 5.8	24.2 ± 8.5	*t* = −5.89	<0.001
CPP (mmHg)	61.8 ± 9.7	65.2 ± 8.1	56.8 ± 9.9	*t* = 5.78	<0.001
PbtO₂ (mmHg)	19.2 ± 5.8	21.5 ± 4.9	15.8 ± 5.2	*t* = 6.89	<0.001
LPR	28.3 ± 9.6	24.1 ± 7.2	34.5 ± 9.1	*t* = −7.85	<0.001
Interventions, *n* (%)					
Any intervention	122 (85.3)	69 (80.2)	53 (93.0)	*χ*^2^ = 4.61	0.032
Hypotensive therapy	58 (40.6)	25 (29.1)	33 (57.9)	*χ*^2^ = 11.98	<0.001
Sedation therapy	65 (45.5)	30 (34.9)	35 (61.4)	*χ*^2^ = 9.68	0.002
Surgical intervention	49 (34.3)	22 (25.6)	27 (47.4)	*χ*^2^ = 7.24	0.007

**Figure 2:**
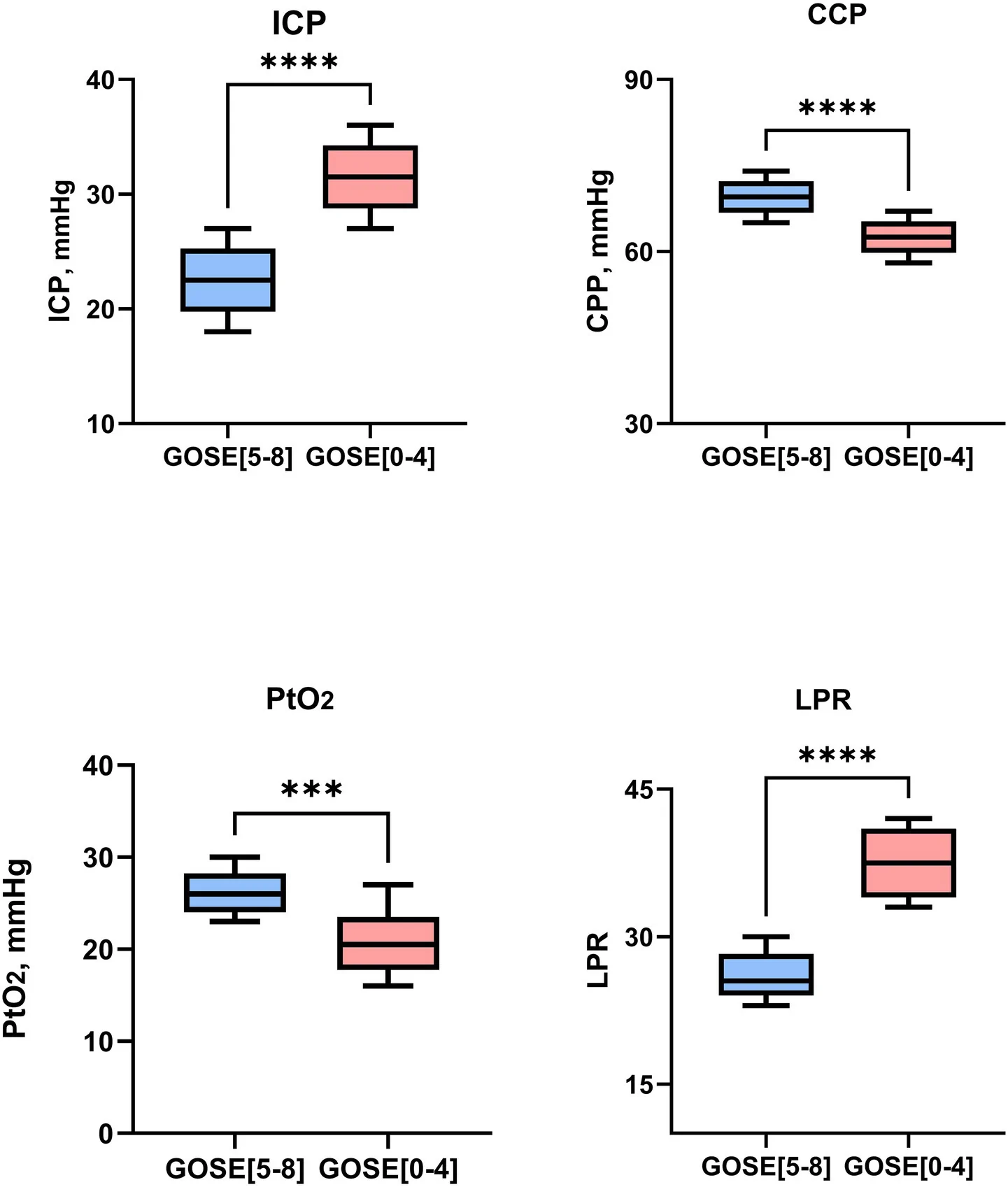
Comparison of multimodal monitoring parameters between favorable and unfavorable outcome groups. Box plots demonstrate the distribution differences of four monitoring parameters (ICP, CPP, PbtO₂, and LPR) between the two outcome groups. The boxes represent interquartile ranges, the horizontal lines within boxes indicate medians, and the whiskers extend to 1.5 times the interquartile range. “*” indicates *p* < 0.05.

Figure 2 illustrates the distribution differences of the four core multimodal monitoring parameters between the favorable and unfavorable outcome groups. Box-plot analysis revealed that the unfavorable outcome group had significantly higher ICP (median: 18.5 vs. 12.2 mmHg, *p* < 0.001) and lower CPP (median: 58.3 vs. 68.7 mmHg, *p* < 0.001) compared to the favorable outcome group. Regarding cerebral oxygenation, the unfavorable outcome group showed significantly lower PbtO₂ levels (median: 15.8 vs. 24.3 mmHg, *p* < 0.001) and elevated LPR (median: 38.6 vs. 21.4, *p* < 0.001), visually demonstrating a significant association between multimodal monitoring parameters and patient outcomes. The unfavorable outcome group exhibited characteristic changes including intracranial hypertension, cerebral hypoperfusion, cerebral hypoxia, and impaired energy metabolism.

**Figure 3:**
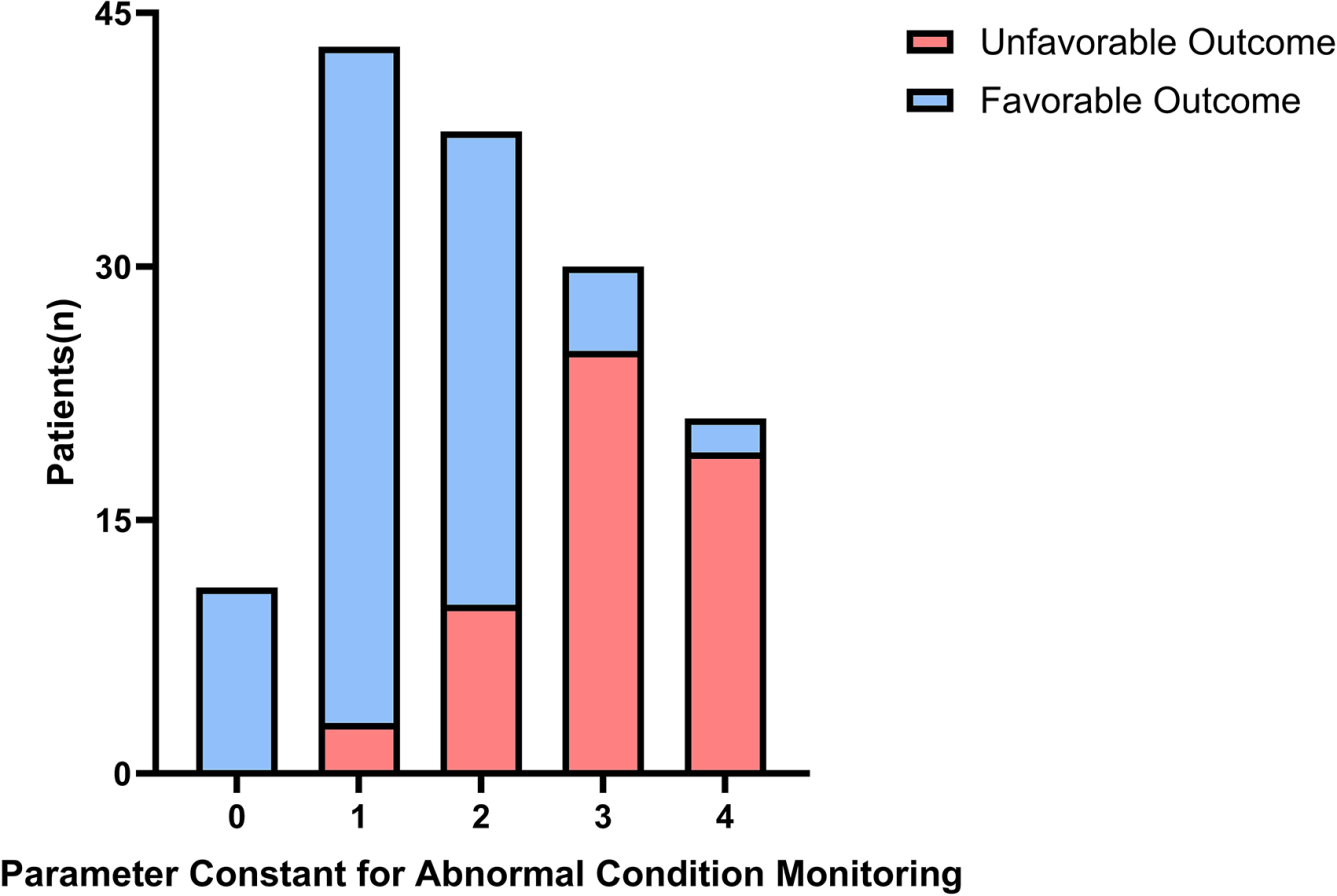
Cumulative effect analysis of the relationship between number of abnormal parameters and clinical outcomes. Stacked bar chart demonstrating the dose–response relationship between the number of abnormal parameters and the risk of unfavorable outcomes. The *x*-axis represents the number of abnormal parameters (0–4), and the *y*-axis indicates the percentage of cases. Blue segments represent the proportion of patients with favorable outcomes, while red segments represent those with unfavorable outcomes.

A stacked bar chart (Figure 3) further revealed a dose–response relationship between the number of abnormal parameters and clinical outcomes. As the number of abnormal parameters increased, the proportion of patients with unfavorable outcomes showed a marked upward trend. When all four parameters were within normal ranges (0 abnormal parameters), the favorable outcome rate reached 85.7%. With one abnormal parameter, the proportion of unfavorable outcomes increased to 32.4%. When the number of abnormal parameters reached two, three, and four, the proportion of unfavorable outcomes increased to 57.1, 78.9, and 91.7%, respectively. This clear gradient trend (*χ*^2^ trend test, *p* < 0.001) strongly suggests a positive correlation between the cumulative number of abnormal parameters in multimodal monitoring and the risk of unfavorable outcomes, providing clinicians with a simple yet effective risk assessment tool.

### 3.3 Multivariate logistic regression analysis

Variance inflation factor (VIF) analysis showed no evidence of significant multicollinearity among the included predictors (all VIF < 5). To control for potential confounding factors, variables with *p* < 0.1 in univariate analysis and all comorbidities were included in the multivariate logistic regression model. The results (Table 3) demonstrated that advanced age, lower admission GCS score, elevated LPR, reduced PbtO₂, and pre-existing coronary heart disease were independent risk factors for unfavorable 6-month outcomes in msTBI patients. Hypertension and diabetes mellitus did not demonstrate independent statistical significance after adjustment.

**Table 3:** Multivariable logistic regression analysis of factors associated with unfavorable outcome at 6 months.

Factor	*β* coefficient	Standard error	Adjusted OR (95% CI)	*p*-value
Age (per 1-year increase)	0.054	0.020	1.055 (1.015–1.098)	0.007
Admission GCS (per 1-point decrease)	−0.338	0.144	0.713 (0.537–0.947)	0.019
LPR (per 10-unit increase)	0.121	0.040	1.129 (1.044–1.220)	0.002
PbtO₂ (per 1-mmHg decrease)	−0.172	0.073	0.842 (0.730–0.972)	0.018
Coronary artery disease (yes vs. no)	1.352	0.691	3.866 (0.998–14.976)	0.050
Hypertension (yes vs. no)	0.842	0.482	2.321 (0.902–5.973)	0.081
Diabetes mellitus (yes vs. no)	0.725	0.521	2.065 (0.744–5.730)	0.164

Variance inflation factors (VIF) for all variables ranged from 1.2 to 3.8, indicating no significant multicollinearity.

### 3.4 Predictive model performance

**Figure 4:**
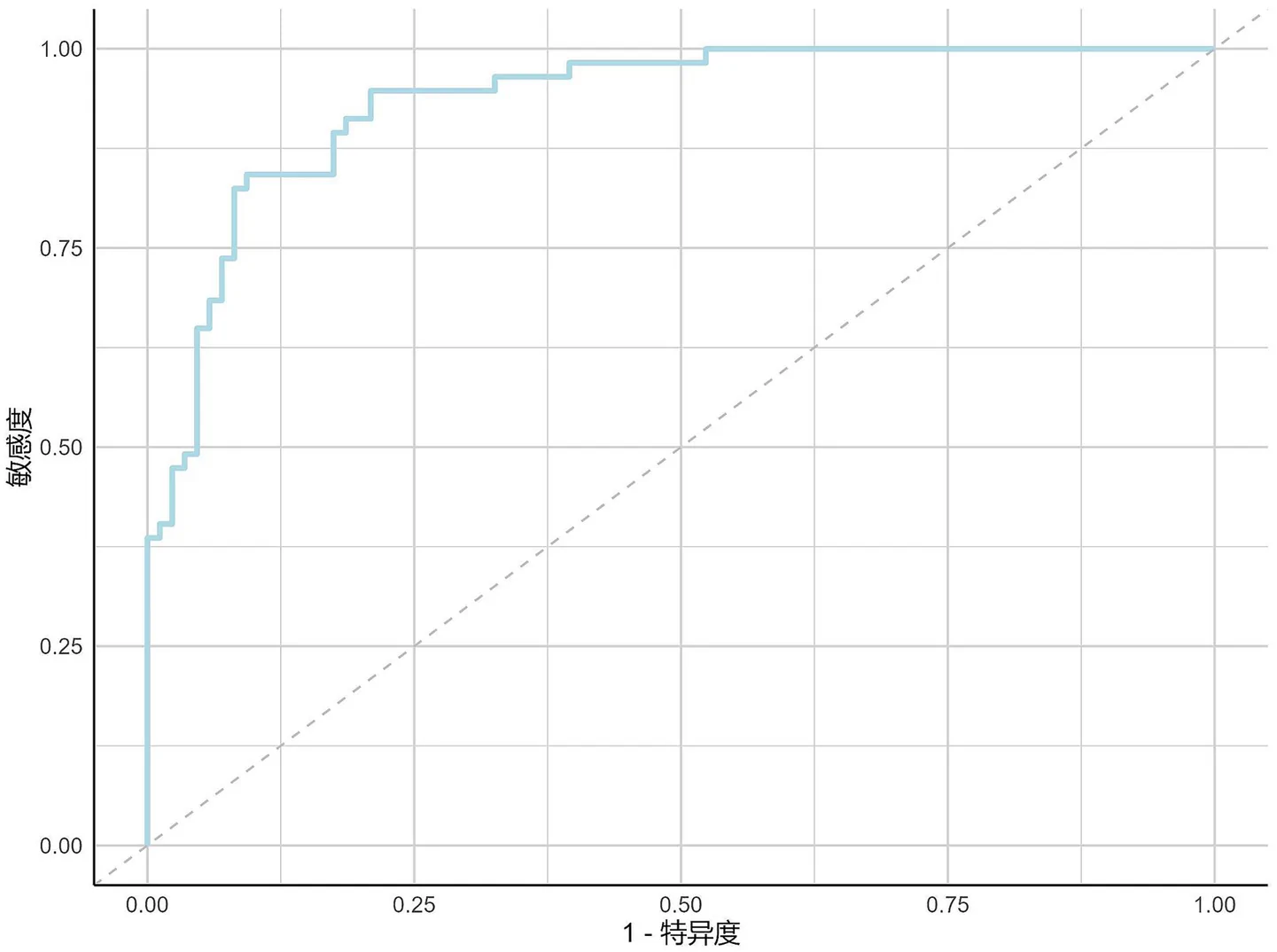
Receiver operating characteristic (ROC) curve of the prognostic prediction model based on multimodal monitoring parameters. (*χ*^2^ = 71.45, *p* < 0.001; Nagelkerke *R*^2^ = 0.528; AUC = 0.882).

The prognostic prediction model constructed based on the aforementioned independent factors demonstrated excellent discriminative ability, with an area under the ROC curve (AUC) of 0.882 (95% CI: 0.820–0.944), as shown in Figure 4. The Hosmer-Lemeshow test indicated good model calibration (*p* = 0.523). To assess the robustness of the model, internal validation was performed using bootstrapping with 1,000 resamples. The optimism-corrected area under the curve (AUC) was 0.861, indicating good model stability.

### 3.5 Subgroup analysis

**Figure 5:**
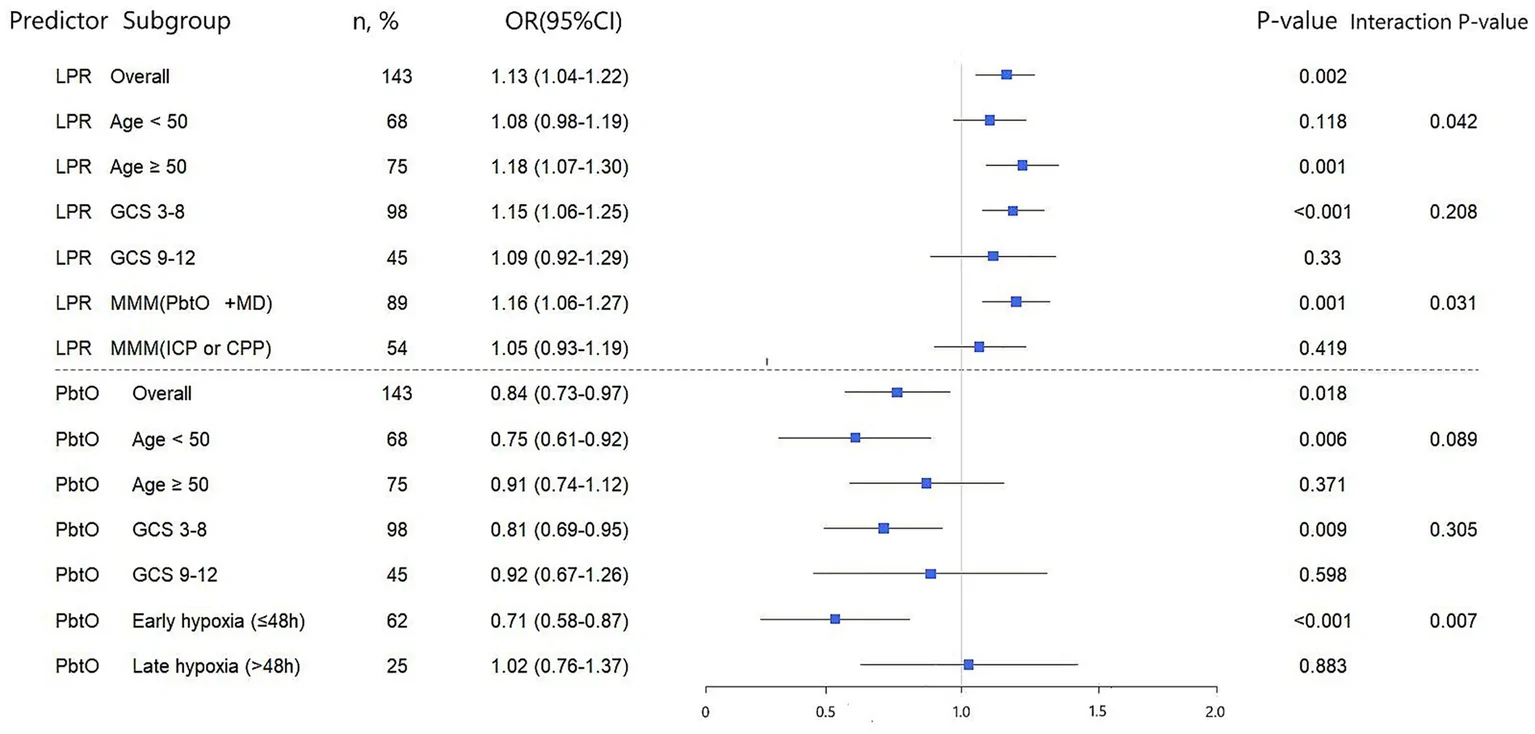
Subgroup analysis of the predictive value of multimodal monitoring parameters.

Subgroup analyses revealed significant heterogeneity in the predictive value of multimodal monitoring parameters (Figure 5). First, age stratification analysis demonstrated that the predictive value of LPR was significantly enhanced in patients aged ≥50 years (OR = 1.18 vs. 1.08, P for interaction = 0.042), suggesting diminished compensatory capacity for metabolic disturbances in the aging brain. Second, the completeness of monitoring modalities significantly influenced predictive accuracy. The LPR showed superior predictive value in the complete multimodal monitoring group (PbtO₂ + microdialysis) compared to the partial monitoring group (OR = 1.16 vs. 1.05, P for interaction = 0.031), further supporting the incremental prognostic value of comprehensive multiparameter monitoring.

Notably, the timing of cerebral hypoxia onset significantly modified prognostic outcomes: early hypoxia (≤48 h) demonstrated a strong association with unfavorable outcomes (OR = 0.71, *p* < 0.001), while late hypoxia (>48 h) showed no significant predictive value (P for interaction = 0.007), emphasizing the critical importance of early intervention. Furthermore, multimodal monitoring parameters exhibited enhanced predictive performance in patients with extremely severe injuries (GCS 3–8), providing an objective basis for prognostic assessment in this critically ill population.

## 4 Discussion

This retrospective cohort study of 143 patients with moderate-to-severe traumatic brain injury (msTBI) systematically evaluated the predictive value of acute-phase multimodal neuromonitoring parameters for neurological functional outcomes at 6 months post-injury. A prognostic prediction model incorporating age, admission GCS score, brain tissue oxygen tension (PbtO₂), lactate/pyruvate ratio (LPR), and history of coronary artery disease was developed. The model demonstrated excellent discrimination (AUC = 0.882) and calibration (*p* = 0.523), significantly outperforming traditional uniphysiological parameter-based assessment systems. This provides robust evidence for the implementation of individualized and precise neurocritical care.

### 4.1 Independent prognostic value of multimodal monitoring parameters

Previous studies have established intracranial pressure (ICP) and cerebral perfusion pressure (CPP) as central metrics in TBI management. However, their limitations in guiding therapy have become increasingly apparent—some patients still experience secondary brain injury and poor outcomes even when ICP/CPP are within target ranges (16–18). This study further confirms that relying solely on ICP/CPP fails to fully capture the essence of cerebral metabolic disturbances. After adjusting for age, comorbidities, and injury severity, elevated LPR and reduced PbtO₂ were identified as independent risk factors for unfavorable outcomes, with effect sizes significantly larger than those of traditional parameters. Specifically, each unit increase in LPR was associated with a 12.9% increase in the risk of unfavorable outcome (OR = 1.13, 95% CI: 1.04–1.22, *p* = 0.002), while each 1 mmHg decrease in PbtO₂ increased the risk by 15.8% (OR = 0.84, 95% CI: 0.73–0.97, *p* = 0.018). These findings underscore the central role of “cerebral metabolic crisis” in secondary injury, suggesting that even under hemodynamically stable conditions, persistent mitochondrial dysfunction or tissue hypoxia can drive neuronal death (19–21).

### 4.2 Incremental prognostic value of multimodal integration

A key finding of this study is the superior predictive performance of comprehensive multimodal monitoring (PbtO₂ + cerebral microdialysis) compared to ICP/CPP monitoring alone. Subgroup analysis revealed that in patients monitored with both PbtO₂ and LPR, the predictive power of LPR for neurological outcomes was significantly enhanced (OR = 1.16 vs. 1.05, P for interaction = 0.031), indicating that multiparameter integrated analysis provides a more comprehensive pathophysiological profile. These results are consistent with previous studies and suggest that integrated monitoring strategies should be prioritized in clinical practice, particularly in well-resourced high-level neurocritical care units, to facilitate a paradigm shift from “single-threshold intervention” to “multidimensional dynamic assessment” (17, 22).

### 4.3 Time-window effect and importance of early intervention

Another critical finding is the significant heterogeneity in the impact of cerebral hypoxia timing on outcomes. Early hypoxic events (≤48 h) were associated with a markedly high risk of unfavorable outcome (OR = 0.71, *p* < 0.001), whereas late hypoxia had no significant predictive effect. This “time-window effect” highlights the importance of the first 48 h post-injury as a golden period for neuroprotection. Early cerebral hypoxia likely reflects acute energy failure and microcirculatory dysfunction following primary injury. If not corrected promptly, it may trigger an irreversible apoptotic cascade. In contrast, late hypoxia is more influenced by systemic factors (e.g., infection, anemia), is more reversible, and has less impact on long-term functional recovery. Therefore, our data strongly support the initiation of multimodal monitoring immediately upon admission to enable targeted interventions within the critical time window.

### 4.4 Modifying effects of age and comorbidities

This study also revealed the modifying effect of age on the prognostic impact of metabolic disturbances. The predictive power of LPR was significantly enhanced in patients aged ≥50 years (P for interaction = 0.042), suggesting reduced compensatory capacity in the aging brain under metabolic stress, making it more susceptible to neurological deterioration due to energy supply–demand imbalance. Furthermore, pre-existing coronary artery disease was identified as an independent risk factor (OR = 3.87, *p* = 0.05), possibly related to impaired cerebral autoregulation due to systemic vascular dysfunction. These findings remind clinicians to more actively apply multimodal monitoring in elderly TBI patients or those with cardiovascular comorbidities to identify high-risk individuals and implement more aggressive metabolic support strategies (23–25).

### 4.5 Clinical translation potential of the model

The prognostic model constructed based on the above independent predictors demonstrated excellent discriminative ability (AUC = 0.882), significantly outperforming classical IMPACT and CRASH models in similar populations (typically AUC between 0.70–0.80) (3, 4). This model incorporates not only biological markers reflecting cerebral parenchymal status (LPR, PbtO₂) but also easily obtainable demographic and comorbidity information, demonstrating good operability and generalizability. Future prospective studies are warranted to further validate its external validity and explore its integration into clinical decision support systems (CDSS) to assist physicians in early risk stratification, treatment intensity adjustment, and family communication.

### 4.6 Study limitations

Several limitations should be acknowledged. First, this study included only patients who underwent complete multimodal monitoring (ICP, PbtO₂, and MD), which may introduce selection bias. Patients selected for advanced neuromonitoring are often those with more severe injuries or better baseline physiological reserve, potentially limiting the generalizability of our model to the broader msTBI population. Second, due to the retrospective observational design, unmeasured confounders—such as variations in sedation depth, hyperventilation protocols, and timing of surgical intervention—may have influenced both monitoring parameters and outcomes. Third, it should be noted that multimodal monitoring parameters are both pathophysiological indicators and targets of therapy. Therefore, their association with outcome may be influenced by the intensity and timing of interventions. For example, a low PbtO₂ value may trigger oxygen optimization or osmotherapy, thereby altering the natural course of injury. Future studies incorporating treatment intensity scores or time-varying covariates may help disentangle these effects. Fourth, although internal validation was performed, external validation in multicenter cohorts is needed before widespread clinical implementation. Finally, the omission of dynamic imaging changes (e.g., CT/MRI progression) or inflammatory markers may affect the comprehensiveness of the model.

## 5 Conclusion

This study confirms that a prediction model incorporating acute-phase multimodal neuromonitoring parameters—particularly LPR and PbtO₂—can effectively identify individuals with moderate-to-severe traumatic brain injury who are at high risk of unfavorable long-term outcomes. Age, coronary artery disease, and the timing of cerebral hypoxia significantly modulated the prognostic value of monitoring parameters. These findings support the adoption of integrated and dynamic monitoring strategies in neurocritical care and lay the groundwork for the development of precise prognostic assessment tools. Future multicenter prospective studies are warranted to validate the generalizability of the proposed model and to explore its practical application in individualized treatment decision-making.

## Data Availability

The raw data supporting the conclusions of this article will be made available by the authors, without undue reservation.
